# Body mass index, not local adiposity, best predicts surgical site infection following surgical fixation of distal femur fractures

**DOI:** 10.1007/s00590-026-04712-2

**Published:** 2026-03-17

**Authors:** Brian D. Rust, Daniel E. Pereira, David W. Barton, Mitchel R. Obey, Christopher M. McAndrew, Marschall B. Berkes, Jenna-Leigh Wilson

**Affiliations:** 1 Department of Orthopaedic Surgery, Washington University School of Medicine, St. Louis, MO USA; 2 Department of Orthopaedics and Rehabilitation, Penn State College of Medicine, Hershey, PA USA

**Keywords:** Distal femur fracture, Open reduction internal fixation, Surgical site infection, Body mass index, Subcutaneous fat thickness, Orthopaedic trauma

## Abstract

**Purpose:**

Body mass index (BMI) is a recognized risk factor for infection in distal femur fracture management, but the role of local subcutaneous fat thickness at incision sites remains unclear. This study evaluated whether local adiposity independently predicts surgical site infection (SSI) after open reduction internal fixation (ORIF) and compared its predictive value to BMI.

**Methods:**

We conducted a retrospective case–control study of 302 adults who underwent ORIF for distal femur fractures at a Level 1 trauma center (2021–2024). Subcutaneous fat depth was measured at lateral, prepatellar, and, when available, medial sites on plain radiographs. The primary outcome was SSI requiring irrigation and debridement within 1 year. Logistic regression assessed the predictive value of fat thickness and BMI, with subgroup analysis of periprosthetic fractures.

**Results:**

Nineteen patients (6.3%) developed SSI. On univariate analysis, greater lateral, prepatellar, medial fat, and BMI were significantly associated with infection. Logistic regression showed lateral and prepatellar fat predicted SSI, but these effects were lost once BMI was included, confirming BMI as the stronger independent predictor. In the periprosthetic subgroup, neither BMI nor adiposity predicted infection.

**Conclusion:**

In distal femur fractures, BMI outperformed local fat thickness as a predictor of SSI, supporting its use as a practical risk stratification tool. While greater local adiposity correlated with reoperation, these associations diminished when BMI was considered. In periprosthetic fractures, no predictors were identified, highlighting the need for larger studies to clarify whether implants alter infection risk.

## Introduction

Distal femur fractures are increasing in incidence with an increasingly aging population. These injuries are typically treated with open reduction internal fixation (ORIF) by using a retrograde intramedullary implant, a lateral or medial locking plate, or a combination of these for fractures around stable femoral components. As these operations are more commonly observed, so are their complications following surgical care. In particular, the infection rate following surgical repair is around 3.6–7% [1–4]. While the literature has shown a connection between surgical site infection (SSI) rates and Body Mass Index (BMI) for native distal femur fractures, it is unclear whether this is a result of the systemic effects of obesity alone versus the proportional increased local adiposity of the distal thigh [2, 4, 5]. Specifically, the subcutaneous fat layer superficial to the lateral femoral epicondyle, the patellar tendon, or the medial femoral epicondyle which are dissected in order to clean, reduce and fix these fractures [6, 7].

Obesity is a chronic disease which has been associated with a host of adverse systemic and postoperative complications in orthopaedic trauma. For example, higher rates of venous thromboembolism, renal complications, perioperative cardiac events, respiratory complications, and short-term mortality have been associated with surgical management of patients with high BMI [8–12]. BMI has also been identified as a modifiable risk factor for surgical site infection for elective procedures [13–15]. The American Academy of Orthopaedic Surgeons (AAOS) has noted that increasing BMI correlates strongly with SSI risk, and this relationship persists after adjustment for other risk factors [16]. However, although obesity is independently correlated with these outcomes, it has been suggested that local fat adiposity may be a greater driver of these outcomes; particularly for patients where subcutaneous fat distribution is variable. For that reason, it has been proposed that BMI is too general of a measure to consider when treating wound complications and infections of the surgical site. In arthroplasty and spine operations, increased subcutaneous fat at the incision site has been associated with an increased chance of developing an SSI [17–19]. Furthermore, there has been literature suggesting that direct measurement of this fat layer shows a stronger independent association with SSI risk than BMI [20, 21]. However, contradictory reports, such as the study on ORIF of acetabulum fractures through a Kocher-Langenbeck approach by Weick et al., have contested the local impact of local adiposity and SSI [22].

Given the relative rate of infection following surgery for distal femur fractures, there is a need for greater risk stratification. For that reason, this study aims to determine if subcutaneous fat depth at the surgical site is predictive of SSI rates for distal femur fracture ORIF and to compare its predictive value to that of BMI. We intend findings to direct intra-operative and postoperative care for patients at risk.

## Materials and methods

### Study design and patient population

A retrospective review was conducted in adult patients diagnosed with a distal femur fracture. Adult (> 50 years old) patients underwent ORIF at a single Level 1, tertiary referral trauma center between January 1, 2021, and December 31, 2024. Institutional Review Board approval was obtained, and patients were identified in the institution’s trauma database using CPT codes (CPT 27506, 27,511, 27,513, and 27,514) and secondarily verified with direct chart review and radiographic verification. All AO 33A/B/C native fractures and Su 1/2/3 periprosthetic fractures were included. Exclusion criteria included patients with a pathologic fracture, open fractures, and patients who received an isolated antegrade intramedullary nail. Fixation constructs included retrograde intramedullary nail, lateral locking plate, nail-plate construct, and dual plate constructs. These were determined according to the sole discretion of the primary surgeon and applied in standard fashion. For antibiotics, all patients were treated with a standard regimen of perioperative cefazolin both prior to incision and for 24 h after surgery unless there was a known allergy.

### Collected variables

**Fig. 1:**
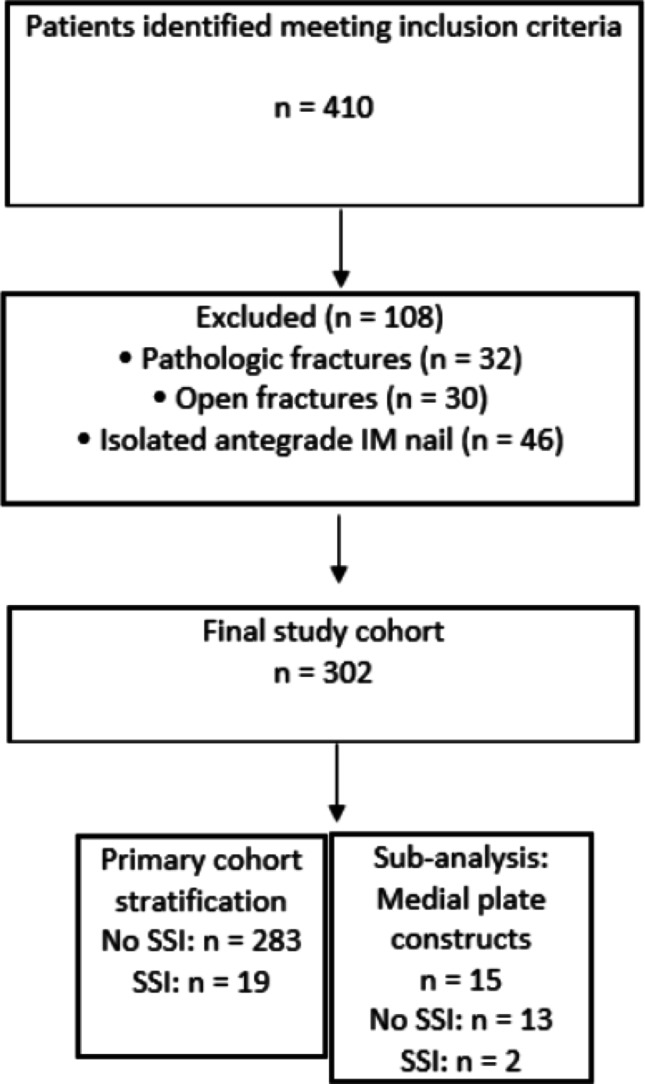
Examples of prepatellar tendon and lateral epicondyle subcutaneous adipose tissue measurements

Surgical site infection was defined based on documentation in the electronic medical record (EMR) of a post-operative infected wound complication which required reoperation for irrigation and debridement (I&D) within 1 year of the original surgery. Axial imaging such as Computed Tomography (CT) is not routinely obtained in this institution for these injuries, for that reason, standard radiographs of the knee or femur were utilized which has been previously employed for similar studies [23–25]. All radiographic measurements were performed using the Sectra Picture Archiving and Communication System (PACS) (Sectra AB, Linköping, Sweden). Pre-operative standard anteroposterior and lateral injury radiographs were reviewed, and measurements were obtained using the calibrated, built-in digital measurement tool within the software. Representative radiographs of a standard AP or lateral of the knee on either knee, femur, or tibial films were utilized to normalize measurements. Subcutaneous fat thickness perpendicular to the skin and the apex of the lateral femoral epicondyle, the apex of the medial epicondyle, or the patellar tendon were collected for every patient at the site of incision. All distances were recorded in millimeters. Representative measurements are demonstrated in Fig. 1.

### Statistical analysis

Data was analyzed using IBM SPSS Statistics Version 27 (IBM Corp., Armonk, NY). Continuous variables were assessed for normality using the Shapiro–Wilk test. Normally distributed variables were compared between groups using independent-samples t-tests, while non-normally distributed variables were compared using Mann–Whitney U tests. Categorical variables were compared using Chi-square tests to determine difference in descriptive data between cohorts.

Univariate analyses were performed to evaluate the association between lateral fat thickness, prepatellar tendon fat thickness, medial fat thickness, BMI, and the development of postoperative infection within 1 year. Binary logistic regression was used to determine the predictive value of these variables, with results expressed as odds ratios (ORs). A multivariable model was constructed to control for potential confounding factors. Because medial fat thickness was only collected for those with a medial plate component of their construct, analyses involving this variable were performed on those patients separately. Bivariate correlation analysis was then performed to determine collinearity among the variables.

Subgroup analyses were performed for periprosthetic fractures and native distal femur fractures using the above process. As there were no periprosthetic infections among those with medial plate constructs, this variable was not included for that sub-group.

## Results

### Patient characteristics

**Fig. 2:**
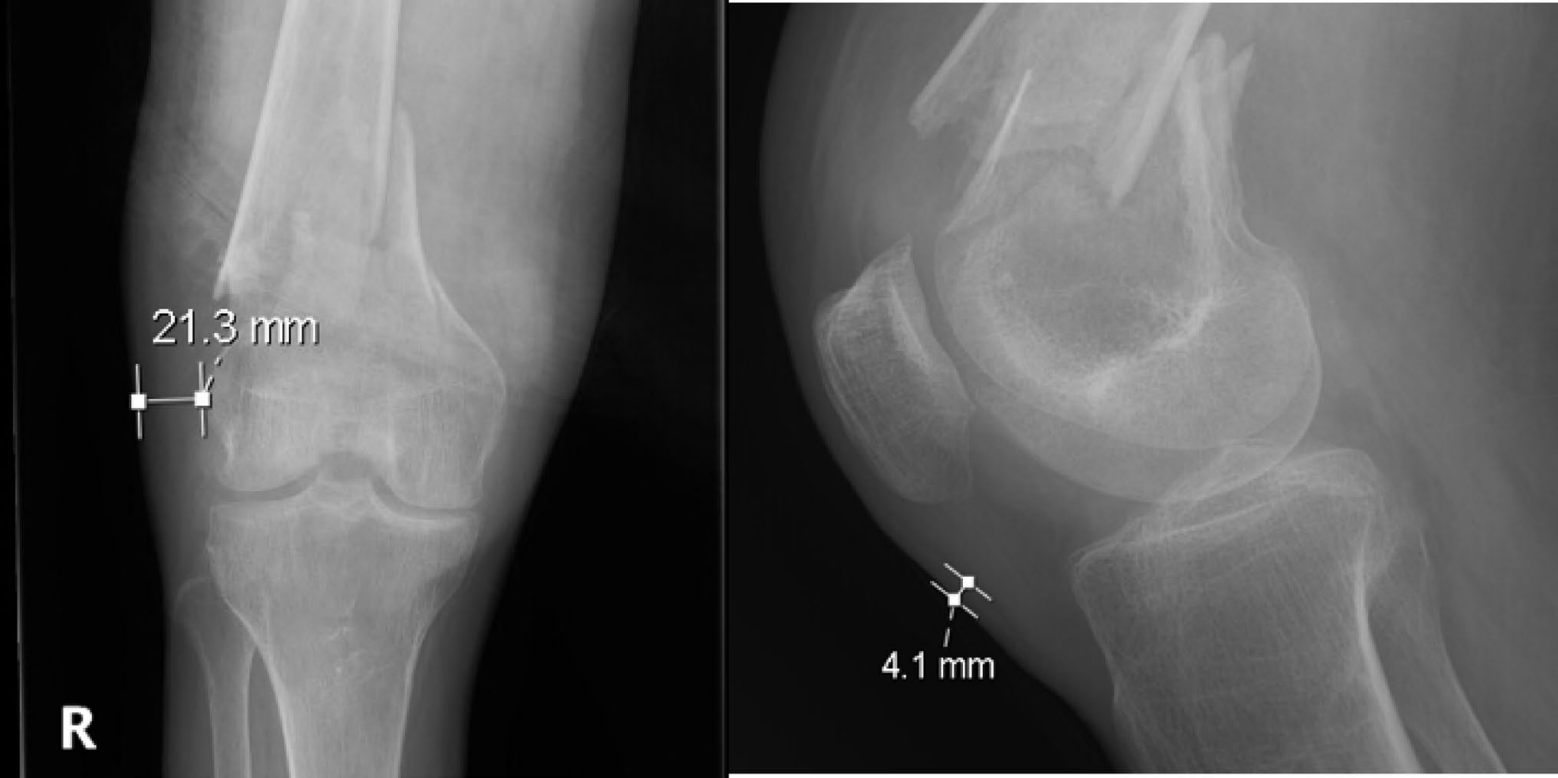
Patient recruitment flowchart

A total of 302 patients were included in the full cohort, of whom 19 (6.3%) developed a postoperative infection requiring reoperation noted within 1 year (Fig. 2). A subset of 15 patients had a medial plate component of their construct, with 2 developing an infection at their medial incision. All infected patients received I&D within 1-year post-op as part of management. The first I&D for the infected group occurred, on average, 85.16 days after the original ORIF. Baseline demographics and comorbidities showed no statistical differences (Table 1). It should be noted that infection rates varied significantly by fixation construct (*p* = 0.030). Pairwise comparisons indicated that dual plate fixation (20%) showed a markedly higher infection rate compared to nail fixation (0%), but all other comparisons lacked significance. Also, those that had an SSI had a median surgical time that was 39 min longer than that of the noninfected cohort (Table 1).

**Table 1:** Patient demographics and comorbidities

		No SSI, *n* (%) (*N* = 283)	SSI, *n* (%) (*N* = 19)	*p*-value
Sex				0.930
	Male	62 (21.9%)	4 (21.1%)	
	Female	221 (78.1%)	15 (78.9%)	
Race				0.886
	White	240 (84.8%)	15 (78.9%)	
	Black or African American	38 (13.4%)	4 (21.1%)	
	Asian	1 (0.4%)	0	
	Pacific Islander	1 (0.4%)	0	
	Not listed	3 (1%)	0	
Age, y, mean (SD)		73.51 (10.77)	68.84 (12.75)	0.112
Insurance				0.492
	Private	27 (9.5%)	1 (5.3%)	
	Medicare	232 (82.0%)	17 (89.5%)	
	Medicaid	18 (6.4%)	0	
	Uninsured	2 (0.7%)	0	
	Other	4 (1.4%)	1 (5.2%)	
Diabetes mellitus				0.673
	Yes	91 (32.2%)	7 (36.8%)	
	No	192 (67.8%)	12 (63.2%)	
Chronic kidney disease				0.174
	Yes	53 (19.1%)	6 (31.8%)	
	No	229 (80.9%)	13 (68.2%)	
Smoking status				0.673
	Yes	39 (13.7%)	4 (21.1%)	
	No	230 (81.3%)	14 (73.7%)	
	Never Assessed	14 (5%)	1 (5.2%)	
ASA score				0.597
	1	1 (0.4%)	0	
	2	53 (18.7%)	2 (10.5%)	
	3	195 (68.9%)	13 (68.4%)	
	4	34 (12.0%)	4 (21.1%)	
Distal femur type				0.697
	Total Native	128 (45.3%)	10 (52.6%)	
	Total Prosthetic	155 (54.7%)	9 (47.4%)	
Fracture classification				0.619
	AO 33A	79 (27.9%)	4 (21.2%)	
	AO 33B	2 (0.7%)	0	
	AO 33C	47 (16.6%)	6 (31.6%)	
	Su 1	40(14.1%)	2 (10.5%)	
	Su 2	82 (29%)	4 (21.2%)	
	Su 3	33 (11.7%)	3 (15.8%)	
Surgical time, minutes, median (SD)		146 (56.37)	185 (50.20)	**0.004**
Construct used				**0.030**
	Nail	58 (20.5%)	0	
	Plate	59 (20.8%)	5 (26.3%)	
	Dual Plate	12 (4.2%)	3 (15.8%)	
	Nail-Plate	154 (54.4%)	11 (57.9%)	
Construct pairwise comparisons	Comparison	*p*-value		
	Nail versus plate	0.059		
	Nail versus dual plate	**0.007**		
	Nail versus nail/plate	0.071		
	Plate versus dual plate	0.171		
	Plate versus nail/plate	0.776		
	Dual plate versus nail/plate	0.097		

Bold values indicate statistically signifi cant p-values

### Normality testing and group comparisons

On univariate analysis, patients who developed infection had statistically significantly higher lateral fat (*p* = 0.003), prepatellar fat (*p* = 0.003), and BMI (*p* < 0.001). For medial subcutaneous fat, analysis showed a statistically significantly higher depth of fat in the infection cohort (*p* = 0.027) (Table 2).

**Table 2:** Fat and BMI by infection status at 1 year

Variable	No SSI Mean (mm) (SD)	SSI Mean (mm) (SD)	*p*-value
Lateral fat	29.935 (14.36)	39.721 (14.08)	**0.003**
Patellar tendon fat	7.696 (5.70)	11.268 (6.64)	**0.003**
Medial fat	37.108 (17.96)	71.950 (0.92)	**0.027**
BMI	31.096 (8.65)	39.484 (8.26)	** < 0.001**
Prosthesis sub-group			
Lateral fat	30.60 (14.43)	39.88 (13.41)	**0.043**
Patellar tendon fat	8.06 (6.07)	10.51 (6.45)	0.153
BMI	31.79 (8.02)	37.27 (8.49)	**0.048**
Native sub-group			
Lateral fat	29.14 (14.29)	39.58 (15.38)	**0.027**
Patellar tendon fat	7.26 (5.22)	11.95 (7.08)	**0.005**
Medial fat	35.88 (18.83)	71.95 (0.92)	**0.042**
BMI	30.26 (9.32)	41.47 (7.95)	** < 0.001**

Bold values indicate statistically signifi cant p-values

The average age was about 4 years younger in the infection group than in the noninfected group; however, this did not reach statistical significance (*p* = 0.112). Categorical variables such as sex (*p* = 0.930), race (*p* = 0.886), insurance type (*p* = 0.492), smoking status (*p* = 0.673), and diagnosis of diabetes mellitus (*p* = 0.673) or chronic kidney disease (*p* = 0.174) were found to be similar in both groups. As for the injuries in each cohort, the ASA score (*p* = 0.597) and fracture classification (*p* = 0.619) were comparable (Table 1). The distribution of patients with and without knee arthroplasty was not significantly different (*p* = 0.697). There were 24 total surgeons whose patients were included in the analysis, and there was no statistically significant difference among them in infection rate (*p* = 0.084).

### Logistic regression and correlation analysis

In logistic regression analysis, lateral fat (OR = 1.039, *p* = 0.006), prepatellar fat (OR = 1.080, *p* = 0.013), and BMI (OR = 1.101, *p* < 0.001) were each independently significantly associated with postoperative infection while medial fat was not (OR = 2,438,528.392, *p* = 0.985) (Table 3). However, when introducing BMI in the multivariable analysis, lateral fat (OR = 1.008, *p* = 0.725) and prepatellar fat (OR = 1.010, *p* = 0.824) were not found to be primary drivers for infection by comparison (BMI OR = 1.089, *p* = 0.006) (Table 4). Similar results were seen for the native distal femur fracture analysis, but this was not seen in those with a periprosthetic fractures. For that analysis, lateral fat (OR = 1.038, *p* = 0.069), prepatellar fat (OR = 1.053, *p* = 0.249), and BMI (OR = 1.082, *p* = 0.054) were all not significantly associated with postoperative infection (Table 3). The full model for all analyses with odds ratios is demonstrated in Tables 3 and 4. Bivariate correlation analysis revealed a significant strong positive correlation between each of the variables (Tables 5, 6).

**Table 3:** Logistic regression results summary

Predictor	B	S.E	Wald	df	Sig. (p)	OR
BMI	0.096	0.026	13.953	1	**.000**	1.101
Lateral fat	0.038	0.014	7.563	1	**.006**	1.039
Patellar fat	0.077	0.031	6.116	1	**.013**	1.080
Medial fat	14.707	56,757.83	0.000	1	.985	2,438,528.392
Prosthesis sub-group						
BMI	0.079	0.041	3.710	1	0.054	1.082
Lateral fat	0.037	0.020	3.297	1	0.069	1.038
Patellar fat	0.052	0.045	1.330	1	0.249	1.053
Native sub-group						
BMI	0.105	0.033	9.894	1	**0.002**	1.111
Lateral fat	0.040	0.019	4.388	1	**0.036**	1.041
Patellar fat	0.109	0.045	5.780	1	**0.016**	1.115
Medial fat	4.042	818.001	0	1	0.996	56.950

Bold values indicate statistically signifi cant p-values

**Table 4:** Logistic regression results summary (multivariable model)

Predictor	B	S.E	Wald	df	Sig. (p)	OR
Lateral fat	0.008	0.022	0.123	1	.725	1.008
Patellar fat	0.010	0.047	0.050	1	.824	1.010
BMI	0.085	0.031	7.637	1	**.006**	1.089
Prosthesis sub-group						
Lateral fat	0.028	0.030	0.852	1	0.356	1.029
Patellar fat	− 0.030	0.065	0.210	1	0.647	0.971
BMI	0.061	0.051	1.437	1	0.231	1.063
Native sub-group						
Lateral fat	− 0.015	0.032	0.212	1	0.645	0.985
Patellar fat	0.075	0.071	1.098	1	0.295	1.078
BMI	0.100	0.040	6.317	1	**0.012**	1.105

**Table 5:** Correlations among lateral fat, patellar fat, and BMI

Variable	Lateral fat	Patellar fat	BMI
Lateral fat	1	**0.674**	**0.566**
	(*p* < .001)	(*p* < .001)	(*p* < .001)
Patellar fat	**0.674**	1	**0.484**
	(*p* < .001)		(*p* < .001)
BMI	**0.566**	**0.484**	1
	(*p* < .001)	(*p* < .001)

**Table 6:** Correlations between BMI and medial fat

Variable	BMI	Medial fat
BMI	1	**0.849**
		(*p* < .001)
Medial fat	**0.849**	1
	(*p* < .001)	

## Discussion

In this case–control study evaluating infection rates requiring reoperation following fixation of distal femur fractures, BMI was found to be a more reliable predictor than local subcutaneous fat adiposity. While increased subcutaneous fat at the lateral and patellar tendon incision sites predicted infection in univariate analysis, their effect was likely driven by elevated BMI. Analysis of medial fat thickness was limited due to a small total sample and only 2 medial infections, limiting generalizability. Therefore, this study further supports BMI as a practical and reliable clinical tool for assessing infection risk. This would be consistent with previous research that established that higher BMI causes systemic risk for SSI though immune dysregulation, microvascular dysfunction, metabolic inflammation, and impaired wound healing [26–29]. Further work is needed to understand both the systemic and local effects of BMI on infection risk in distal femur fractures.

Infection following surgical fixation can often necessitate a reoperation for debridement and irrigation and possibly further staged surgeries, increasing the burden in an already fragile patient population. For that reason, minimally invasive plate osteosynthesis (MIPO) techniques have been proposed as the mainstay of treatment to reduce the amount of soft tissue damage and stripping [7, 30, 31]. These techniques have been traditionally utilized for the distal lateral locking plate constructs which often use a limited lateral incision at the lateral knee and smaller percutaneous incisions along the axis of the femur. However, with the advent of dual implant techniques to promote early weight bearing and biomechanical survivability, multiple incisions may be made in the medial knee and prepatellar region for a dual plate or nail plate combo, respectively. While these implants have resulted in better systemic and mechanical outcomes, little is known on the local approach-related risk factors related to surgical wound infections.

Previous literature has supported the association of local adiposity measurements estimating surgical risk; even over standalone BMI values [20, 21]. However, in other studies, as arthroplasty or pelvic trauma, surgical site infection (SSI) has not been significantly tied to local subcutaneous fat thickness [22, 24, 32]. Notably, Weick et al. came to this conclusion for acetabular fractures through a Kocher–Langebeck approach using a similar methodology as our own [22]. The underlying assumption is the differential proportion of subcutaneous fat distribution in different anatomical locations. As findings seem to differ between surgical location, approach, and study cohort, it is possible that local adiposity cannot be assumed to be an independent predictor of SSI universally. Our findings reinforce this possibility, showing that local fat thickness alone was not the most predictive factor once BMI was considered. This suggests that previous conflicting results may reflect variability in study design and differences in how adiposity was measured; in addition to how BMI was addressed as a potential confounder. Importantly, the strong correlation between BMI and local fat makes it difficult to separate their effects. Despite BMI being the stronger predictor in our analysis, it remains unclear whether systemic or local adiposity can be understood and treated separately in order to mitigate infection risk. Further study is necessary to understand whether local wound management techniques may mitigate the emergence of infection, especially for individuals in which the correlation between higher BMI and thicker local adiposity is not seen.

Our overall infection rate of 6.3% is consistent with the range of around 3.6–7% as cited in the literature [1–4, 33, 34]. There were differences seen in infection rate between dual plate constructs and retrograde intramedullary nail, but these results should be interpreted cautiously given the small sample found in the dual plate cohort. For the periprosthetic sub-group analysis, the overall infection rate was 5.5%, which is less than the cited rate of 7–7.3% [35, 36]. While it is not impossible that institution-specific factors allowed for a lower infection rate, it is much more likely that our sample was undersized given that this cohort consisted of only 164 patients. Therefore, this infection rate should be interpreted with caution. This study included both native and periprosthetic distal femur fractures. While we expected the periprosthetic cohort to have an elevated risk of infection given a secondary surgery and the presence of indwelling implants, sub-group analysis did not identify any predictors for infection, including BMI. Our findings align with previous reports which demonstrate that while increasing BMI has been indicated as a predictor for overall reoperation, it was not identified as a significant predictor for reoperation as a result of infection [37]. These findings are unexpected, as the presence of an indwelling implant has been independently associated with an increased SSI risk in other orthopedic operations [38]. These results may be a reflection of inadequate power rather than a true difference, but possible confounders may include the presence of a prior surgical scar, the specific implants used, or the altered vascularity of the surrounding soft tissues. While our findings raise the possibility of a periprosthetic paradox, it must be acknowledged that further study is needed before any such phenomenon can be confirmed. A larger, adequately powered study directly comparing native and periprosthetic fracture cohorts would be required to determine whether the presence of a pre-existing total knee arthroplasty influences the effect of BMI on SSI rates.

Of note, there was a difference in surgical length observed between the SSI and noninfected cohorts. A median difference of 39 min was found to be statistically significant. Reasons for this may be due to more complicated fracture morphology, variable experience of the primary surgeons, or the use of a case for resident education and training. It has been shown that increased distal femur ORIF time is associated with increased chances of SSI [3, 5]. This phenomenon is not unique to distal femur fractures and has been reported in other orthopaedic and general surgeries as well [39–41]. It is believed that this may be due to increased exposure to airborne pathogens and greater opportunity for sterile technique violations [42]. In a recent multicenter retrospective study, it was found that each hour in distal femur ORIF increased odds of infection by 15% [3]. While the surgical length difference we observed was not large, it is important to recognize.

This study has several limitations. As a retrospective data analysis, there was no control over how the patient data was collected, and there is a risk of unknown confounders affecting the results. Examples include provider-specific variations in care such as incision length, patient adherence to post-operative instructions, patient medication and drug use history, and the differences in patient home environments. Secondly, the variability of axial imaging modalities, such as CT or MRI, may have affected accuracy. Notably, this study utilized only one data collector for imaging measurements, and both intra- and interobserver reliability were not directly assessed. Of note, while it is the practice at this institution to administer local antibiotics into these wounds, it is not known if this practice was standardized across the entire cohort. As stated before, statistical power was limited for subgroup analyses, particularly for medial fat thickness and the periprosthetic cohort, which restricts the conclusions that can be drawn. Finally, as this was a single institution analysis, there may be an institutional bias affecting infection rates. Within our institution, there was no significant difference among surgeons in infection rate, but this does not completely rule out individual bias that may be hidden by the sample size. Further study is needed to understand the patient-specific drivers for infection and whether these can be mitigated by the orthopaedic surgeon intra- or post-operatively. Also, this population would benefit from expanding the scope of this study to analyze the connection between SSI rates and other variables such as union rates.

## Conclusion

In this large case–control retrospective of distal femur fractures, local subcutaneous fat thickness was correlated with reoperations to address infection in univariate analyses. However, in multivariate analysis, these effects were diminished when BMI was included, reflecting overlap and collinearity between the two measures. These results reaffirm BMI as a convenient and effective tool for infection risk assessment in these patients as opposed to local measurements of fat distribution. In periprosthetic fracture management, BMI and local adiposity were not associated with reoperations as a result of infection, raising the possibility of a unique risk profile. Large comparative studies are required to determine whether the presence of a pre-existing implant truly modifies infection risk factors and to establish whether wound management strategies can mitigate these complications.

## Data Availability

Data is protected health information and is not publicly available, but it can be made available by the authors upon reasonable request.
